# Smelt was the likely beneficiary of an antifreeze gene laterally transferred between fishes

**DOI:** 10.1186/1471-2148-12-190

**Published:** 2012-09-25

**Authors:** Laurie A Graham, Jieying Li, William S Davidson, Peter L Davies

**Affiliations:** 1 Department of Biomedical and Molecular Sciences, Queen’s University, Kingston, ON, K7L 3N6, Canada; 2 Department of Molecular Biology and Biochemistry, Simon Fraser University, Burnaby, BC, V5A 1S6, Canada

**Keywords:** Ice-binding, Horizontal gene transfer, Antifreeze protein, *Osmerus mordax*

## Abstract

**Background:**

Type II antifreeze protein (AFP) from the rainbow smelt, *Osmerus mordax*, is a calcium-dependent C-type lectin homolog, similar to the AFPs from herring and sea raven. While C-type lectins are ubiquitous, type II AFPs are only found in a few species in three widely separated branches of teleost fishes. Furthermore, several other non-homologous AFPs are found in intervening species. We have previously postulated that this sporadic distribution has resulted from lateral gene transfer. The alternative hypothesis, that the AFP evolved from a lectin present in a shared ancestor and that this gene was lost in most species, is not favored because both the exon and intron sequences are highly conserved.

**Results:**

Here we have sequenced and annotated a 160 kb smelt BAC clone containing a centrally-located *AFP* gene along with 14 other genes. Quantitative PCR indicates that there is but a single copy of this gene within the smelt genome, which is atypical for fish *AFP* genes. The corresponding syntenic region has been identified and searched in a number of other species and found to be devoid of lectin or *AFP* sequences. Unlike the introns of the *AFP* gene, the intronic sequences of the flanking genes are not conserved between species. As well, the rate and pattern of mutation in the *AFP* gene are radically different from those seen in other smelt and herring genes.

**Conclusions:**

These results provide stand-alone support for an example of lateral gene transfer between vertebrate species. They should further inform the debate about genetically modified organisms by showing that gene transfer between ‘higher’ eukaryotes can occur naturally. Analysis of the syntenic regions from several fishes strongly suggests that the smelt acquired the *AFP* gene from the herring.

## Background

**Figure 1:**
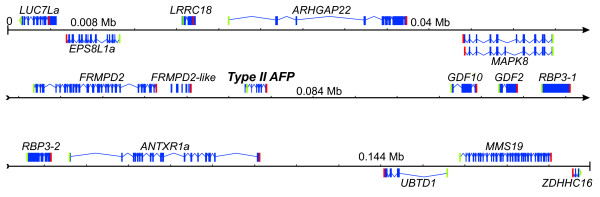
**Annotation of the smelt BAC clone.** Exons are indicated with blue boxes, introns by bent lines and genes encoded by the complementary strand are shown below the line. Start and stop codons are indicated by green and red bars, respectively (or bent bars for incomplete genes). Genes are named using HGNC compatible names (http://www.genenames.org/) with those that do not share conserved synteny with humans denoted with the suffix ‘a’. The locally duplicated copies of *RBP3* are denoted *RBP3-1* and −*2*. Two splice variants are predicted for MAPK8. Note that small exons cannot be resolved at this scale. The annotated sequence has been deposited under GenBank Accession JQ514278 and the genes are described in Additional file 4.

Some organisms that are unable to avoid subzero temperatures use antifreeze proteins (AFPs) that bind to ice and lower the non-equilibrium freezing point by a non-colligative mechanism [1-3]. This allows the organism to survive in a supercooled state in the presence of ice. AFPs have now been reported from disparate branches of the tree of life, including bacteria, fungi, diatoms, plants, insects and fish [3-8]. The structures of these proteins (even within one kingdom) are proving to be as diverse as their taxonomic distribution, but most have repetitive sequences and structures with a flat surface that binds to ice. In fish, four different classes of AFPs provide freezing resistance to their hosts [9]. Two are linear and repetitive; the alpha-helical type I AFPs and the antifreeze glycoproteins (AFGPs), whereas the type II and type III AFPs are globular. The scattered distribution of these AFP types, which often defies taxonomic conventions, has been noted on many occasions and Figure 1 of the 1998 review by Cheng nicely demonstrates this conundrum [10].

The type II AFPs, which are the focus of this study, are derived from the carbohydrate-recognition domain of calcium-dependent (C-type) lectins [11]. Those from herring (*Clupea harengus*) and rainbow smelt (*Osmerus mordax*) retain this calcium dependence, whereas those of the sea raven (*Hemitripterus americanus*) and longsnout poacher (*Brachyopsis rostratus*) do not [11-13]. The structures of herring and poacher AFPs were determined by X-ray crystallography [13,14] and that of the sea raven AFP by NMR [15]. They all contain five disulfide bridges, one to three more than found in other members of this lectin family, and are much more similar to each other than to any other family members [14,16]. This led to the proposal that an ancestral AFP had arisen prior to the separation of the herring and smelt lineages [14], estimated to have taken place well over 200 million years ago [17-19]. The absence of this *AFP* within the genomes of intervening species was attributed to gene loss. However, several lines of evidence, discussed later, indicated to us a different scenario in which the gene was acquired via lateral gene transfer [16].

Type II AFP transcripts were cloned from both closely related and distantly related fish, leading to the curious observation that the AFPs of the rainbow smelt and the Japanese smelt (*Hypomesus nipponensis*, same family) are no more similar to each other than they are to the AFP of the herring (different superorder) [11,16,20]. Previously, we cloned the *AFP* genes of rainbow smelt and herring, and found that the high similarity observed in the coding regions extended to the intronic sequences, which were up to 97% identical. The rate of non-synonymous substitution was not significantly lower than for synonymous substitution, suggesting that strong selection could not explain this similarity. In addition, hybridization bands were only detected for herrings, smelt and sea raven when the DNA of a variety of species was subjected to Southern blot analysis. Together, this evidence suggested that the *AFP* gene was laterally transferred between fish species [16].

The amplification of advantageous genes is thought to be a common adaptive response to rapid environmental changes, such as those that occur at the onset of an ice age [21]. This process has been clearly demonstrated in both bacteria and yeast subjected to other stresses [22,23]. Gene duplication is many orders of magnitude more frequent than advantageous point mutations, although reversion to a single copy may occur once the genes are optimized for their new function [24]. Not surprisingly, the vast majority of AFP-producing organisms contain multiple copies of their *AFP* genes, usually encoding a number of different isoforms. In this, fish are no exception. There are more than 40 gene copies in both winter flounder (type I AFP) [25] and different pout species living near either pole (type III AFP) [26,27]. In the case of AFGPs, they have arisen at least twice via convergent evolution and in both cases, not only are there multiple copies of the genes, but they encode polyprotein precursors that are processed to yield up to 46 mature AFGPs [28]. The type II gene is also amplified in herring, as multiple bands were evident when the DNA from a single individual was subjected to Southern blotting. However, only a single band was evident with rainbow smelt DNA [16].

Lateral (or horizontal) gene transfer (LGT) is a well-accepted phenomenon in unicellular organisms and is particularly evident when bacteria acquire genes for antibiotic resistance [29]. For multicellular organisms the transfer must be to the germ line and the mechanisms (transposons/viruses excepted) are not as obvious as for prokaryotes. We previously postulated that gene transfer may occur between fish during spawning [16], as fish release huge quantities of sperm into the water at this time [30]. Indeed, sperm-mediated transformation is an established laboratory procedure for making transgenic organisms, including fish [31]. Recently, other studies have supported LGT to metazoans [32], including the transfer of carotenoid biosynthetic genes from fungi to aphids [33] and cellulase genes from bacteria to nematodes [34].

The *AFP* genes in herring are likely dispersed and polymorphic as multiple bands were previously observed on a genomic Southern blot, whereas the single band observed in rainbow smelt suggested either a single-copy or a tandemly-duplicated gene [16]. To explore this locus within the genome, we screened a rainbow smelt bacterial artificial chromosome (BAC) library (CHORI-74, [35]) for *AFP* genes. One of 18 overlapping BAC isolates was sequenced in its entirety and the single copy status of the *AFP* gene in the smelt genome was confirmed by quantitative PCR. Syntenic comparisons of the genes flanking the smelt *AFP* were made to sequenced fish genomes. The results remain consistent with and strongly reinforce the hypothesis that the smelt *AFP* gene was acquired via LGT.

## Results

### BAC screening, sequencing and annotation

Eighteen BACs from the smelt library of 52,410 clones (CHORI-74 [35]), representing an 11-fold coverage of the genome, were hybridization positive for an oligonucleotide probe designed from the sixth exon of the smelt *AFP* gene (DQ004949, Table 1). All 18 clones gave a 255 bp PCR product using the probe and an AFP specific primer (Table 1). Semi-quantitative PCR did not show a difference in *AFP* gene copy between clones (data not shown), so three were chosen at random for further analysis. The combined length of the *Bgl*II restriction fragments of clone O0139C19 was greatest. Over 1100 shotgun clones of O0139C19 were Sanger end-sequenced, with an average read length of ~740 bp, providing 5-fold coverage of the BAC insert. However, numerous regions were recalcitrant to standard sequencing methodologies, including simple repeats, such as (GT)_99_, inverted repeats and homopolymeric stretches. Therefore, sequencing primers designed close to these regions were used in reactions employing alternate chemistries. The entire 160 kb sequence was finished, with at least two-fold coverage in problematic areas.

**Table 1:** Probe and primer sequences

**Name**	**Sequence**
*Type II AFP* probe	GCATCCATACACAGCCTTGAAGAGTATGCGTTTG TTAAGG
*Type II AFP* R	CGCACCAGTCAGTAAAATCC
*AFP* (80) qF2	CCATACACAGCCTTGAAGAG
*AFP* (80) qR2	AATCTGAGCCTCCAATCC
PBAC-GMR primer F2	CTATAACCAGACCGTTCAGC
PBAC-GMR primer R2	CTATCCCATATCACCAGCTC
Smelt-*LDH-A* qF2	AACTCCAAGGTGGTGGTG
Smelt-*LDH-A* qR2	GGGACTGTACTTGACGATGT
Smelt-*LUC7La* qF1	AGGAGATCGGGAAGCTACT
Smelt-*LUC7La* qR1	CTGCGAACTTTCTCCACTT
O0139C19 SP6 F	GTGTATCTTTGCCAGCCTAC
O0139C19 SP6 R	AACGTCAACATGGAGGAGTA
O0139C19 T7 F	CACGTCTGGATAGGTGAGAT
O0139C19 T7 R	GAGAGATTGACCTGCACTGT
*RBP-3-2* 5′ inner	GAYGTNTTYGARAAYAAYATHGG
*RBP-3-2* 3′ inner	ACYTTRTTCCANACRTGYTCNAC
*RBP-3-2* 5′ outer	ATHGCNGARGAYGTNGCNTAYTG
*RBP-3-2 3*′ outer	TCRAARAARTANGARCARAANCC
*RBP-3-2* 5′ herring	TCGGAGACTTTGAGCAGGTG
*RBP-3-2* 3′ herring	TCACCTGCTCAAAGTCTCCG

A minimum tiling path was built for the 18 BACs using the BAC end sequences of O0139C19 as sequence tag sites (Additional file 1: Figure S1A). All but one was PCR positive using primers specific for the 3′ of this BAC (Table 1, O0139C19_T7_F & R), indicating that they contained the same *AFP* gene and came from the same region of the rainbow smelt genome. The remaining BAC (O0059H07) was positive for the 5′ end. As the *AFP* gene was centrally located in the sequenced BAC, the biased tiling path was a concern. So, the restriction fragment distributions of the three clones (O0068I08, O0119M24, O0139C19) were analyzed. Their *Bgl*II restriction fragment patterns were highly similar with 25 or more discernible bands (Additional file 2: Table S1). The *in silico Bgl*II digest of the assembled sequence was in complete agreement with the observed restriction pattern of the sequenced clone O0139C19 (Additional file 2: Table S1). Clones O0119M24 and O0068I08 shared all but the fragments found in the first ~10 and ~40 kb, respectively. End sequencing revealed that O0119M24 begins at the *Eco*RI site at base 6274 (*Eco*RI was used to generate these BAC clones via a partial digest of smelt genomic DNA), whereas O0068I08 began at the site at base 42446. Clone O0059H07, which overlapped at the 5′ end of the sequenced clone, had the expected unique 5′ end and terminated at the *Eco*RI site at base 93176. The dearth of clones extending upstream of the sequence insert is likely a result of the unequal distribution of *Eco*RI sites as 11 of the 28 sites are found within the first 25 kb of this 162 kb clone (Additional file 1: Figure S1B), which would decrease the odds of recovering clones spanning this section.

### BAC clone O0139C19 contains fifteen genes

The insert contains a total of fifteen genes, six upstream and eight downstream of the *AFP* gene, with the two terminal genes (*LUC7La*) and end (*ZDHHC16*) being incomplete (Figure 1). These genes range in length from 1.25 to 18 kb, and eleven are on the same strand (top) as the *AFP*. ESTs recovered from other species suggest that the *MAPK8* gene is alternatively spliced. These smelt genes have been named according to their human orthologs as given by the HUGO Gene Nomenclature Committee (http://www.genenames.org/).

Annotation of the smelt BAC insert was aided by the large number of fish ESTs in the database, and the only gene that could not be accurately predicted was *FRMPD2*, just upstream of the *AFP* gene. A total of 33 exons were identified but several of those near the 3′ end of the gene are similar to earlier exons and are annotated separately as *FRMPD2-like*. This may represent a local duplication event but this is difficult to determine as this gene is also poorly annotated in other fish genomes. The other genes contain 1 to 31 exons and encode a variety of proteins with generally unrelated and poorly characterized functions as described in the UniProt database [36]. For example, little is known about UBTD1 other than it contains a single ubiquitin-like domain. The best characterized is RBP3, which is essential for vision as it shuttles retinoids between the retinal pigment epithelium and the photoreceptor cells.

### Quantitative PCR indicates that AFP is a single-copy gene in smelt

The similarity in the restriction fragment patterns of the three BACs mentioned above, as well as the presence of only a single copy of the *AFP* gene on the sequenced BAC, suggested that there is but a single copy of the *AFP* gene in smelt. To test this, qPCR was performed by amplifying portions of the *AFP* gene, *LDH-A* (which is a single copy gene in smelt [37]), *LUC7La* (which is present as a single copy near the *AFP* in BAC O0139C19) and the BAC vector (pBAC-GMR) using the primers listed in Table 1. When DNA from BAC O0139C19 was used as the template, the ΔCT values for each of the serial dilutions were the same for the BAC vector, *AFP* and *LUC7La*, indicating that each was present in a 1:1:1 ratio in the BAC clone. Similar results were obtained when smelt genomic DNA was used as template with a 1:1:1 ratio of *AFP*: *LUC7La*: *LDH-A* (Additional file 3: Table S2). Thus the *AFP* gene of smelt is single copy.

### The dearth of polymorphisms in smelt ESTs is consistent with a single-copy AFP gene

BLAST searches were performed on both the non-redundant and EST databases of NCBI. In addition to one *AFP* gene and two AFP mRNA sequences, 406 ESTs out of almost 37,000 smelt ESTs in the database coded for the AFP. This represents just over 1% of the total. However, only six of those AFP sequences are found in the close to 33,000 ESTs derived from mixed tissues (brain, kidney and spleen [35]), so 10% of the ~4,000 liver transcripts encode the AFP. The liver libraries were derived either from an unspecified number of individuals [35] or from six and nine individuals respectively ([38] and GenBank Accession GR557461, for example)). For Atlantic herring, there are 397 ESTs in the database, derived from the livers of nine individuals [39]. Of these, 34 (~9%) encode the AFP, along with one gene and four mRNA sequences. Transcriptome sequencing of the Pacific herring has generated a large number of additional *AFP* sequences [40] and the assembled *AFP* sequence from liver library was over 99% identical to the Atlantic herring genomic sequence (Genbank accession DQ003023.1).

**Figure 2:**
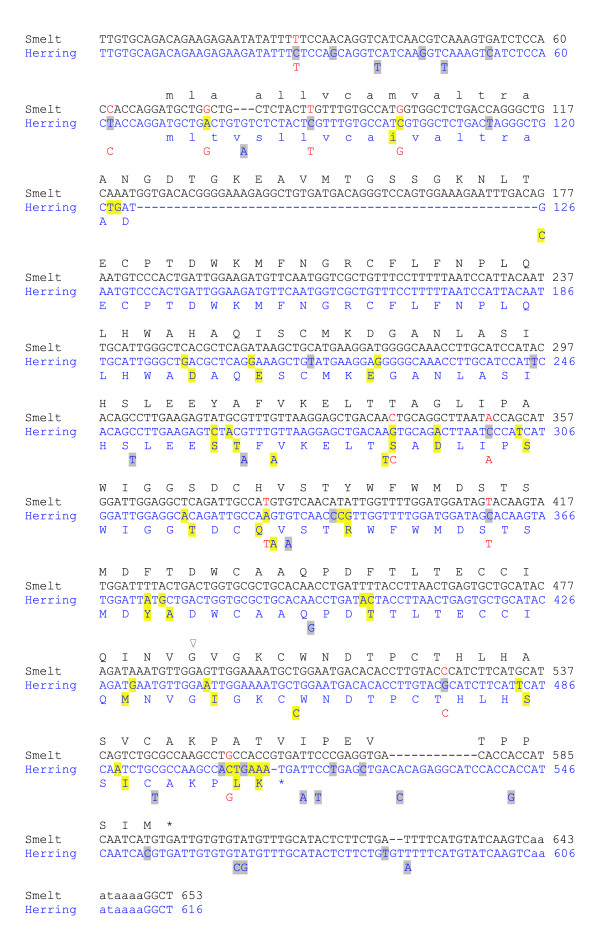
**Alignment of the cDNA sequences of smelt and herring with their deduced protein sequences.** Polymorphisms found in >5% of the Atlantic herring ESTs are shown below the herring sequence (GenBank S65819.1). Differences relative to the smelt sequence (GenBank M96154.1) are highlighted yellow (missense) or gray (silent or non-coding). The only significant polymorphism in smelt is a single base insertion, indicated with an open triangle. The signal peptide and polyadenylation signal are in lower case font. The eleven polymorphisms in the herring ESTs that match the smelt cDNA are in red font. Sequences have been trimmed to remove linker sequences and slight variations in the site of polyadenylation.

Sequence alignments were generated (not shown) for the Atlantic herring and smelt sequences from above and scored for polymorphisms relative to the deduced cDNA sequence from the smelt BAC insert and the longest herring cDNA available (Figure 2). Since the majority of the sequences were derived from lower-quality data (ESTs), only sites containing the same variation in over 1% of the smelt sequences or over 5% (three or more) of the herring sequences were scored. By these criteria, there are 31 (5%) polymorphic sites for herring and only one for smelt; a base insertion causing a frameshift that is found in 24 (6%) of the sequences. This is consistent with the results of the quantitative PCR experiments, which suggests that smelt contains but one copy of the *AFP* gene. There are 47 (8%) differences (gaps excluded) between the smelt and herring sequences, 11 of which are polymorphic in herring, and in all 11 cases, the variation matches that found in smelt. The 5′ and 3′ untranslated regions are also well conserved between species, with identities of 94% (63/68) and 98% (56/57), respectively.

### The smelt AFP gene has had little time to diverge

**Figure 3:**
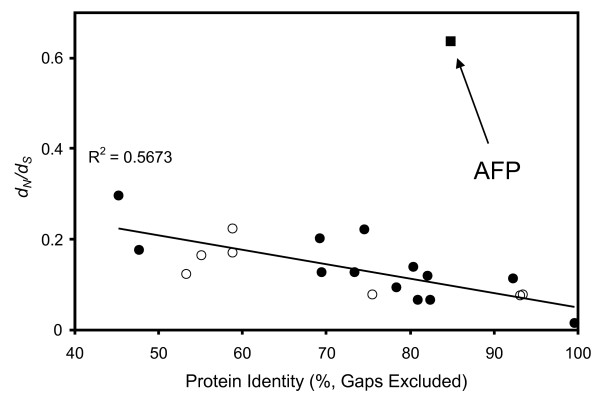
**Mutational analysis of smelt and herring genes.** Ratio of missense changes per missense site to silent changes per silent site (*d*_*N*_/*d*_*S*_) between orthologs (closed circles), paralogs (open circles) and the *AFP* gene (closed square) found in herring and smelt.

We compared the ratios of mutations that change residues (dN) with those that are silent (dS) in 20 genes for which we could find orthologs in both herring and smelt (Figure 3). For 19 of these 20 genes the ratios plotted against percent sequence identity fell close to a straight line. The only exception was the AFP sequence, which was a factor of ~4 removed (above) the line. This discrepancy was a result of a very low rate of change at the silent sites (a more detailed description is available in Additional file 4). The logical interpretation is that the *AFP* gene has not had as much time as the other 19 genes to accrue mutations, again consistent with LGT between the two species long after they diverged.

The homologs used above were also used to determine overall codon usage within each species (data not shown). There were no striking or consistent differences between herring and smelt (data not shown), nor between these two species and fugu (*Takifugu rubripes*) [41]. Therefore, the codon usage pattern of the *AFP* gene was of no use in determining the source species.

### The AFP gene is absent from conserved syntenic regions of other fishes

**Figure 4:**
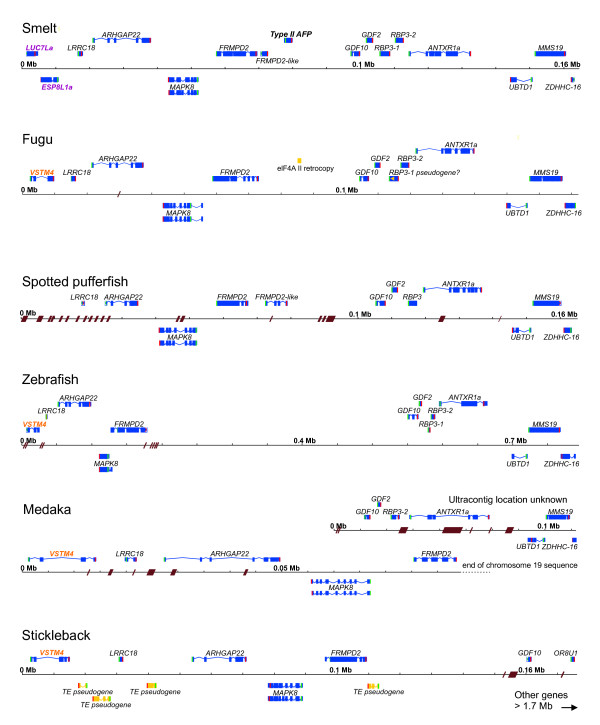
**Apollo visualization of the annotation of the corresponding syntenic regions of other fishes to that of the BAC region containing the*AFP*in smelt.** Features are denoted as in Figure 1. Brown hash marks or parallelograms across the scale line indicate the location and extent (if known) of gaps in the sequence. Genes indicated with orange and purple labels are discontiguous between smelt and the other species. Some repetitive DNAs, such as retrogenes or transposable elements (TE) are indicated with dark yellow boxes and aberrant coding sequence by a yellow arrowhead. Fine details, such as short introns, cannot be resolved at this scale.

Detailed comparisons were made between the smelt BAC sequences and the genomes of those fishes with publicly available genome sequences. Multiple homologs were found for most of the genes, but the conserved syntenic regions were easily identified both by the higher similarity between the genes and the similar gene arrangements (Figure 4). The conserved syntenic regions were downloaded from Ensembl and the genes were reannotated, when necessary, using EST sequences and/or by doing detailed comparisons between the sequences of the six species. As in smelt, there are many stretches of repetitive sequence, some of which give rise to gaps within the assemblies. A detailed analysis of these genome regions is provided in Additional file 4 to demonstrate that the annotation of these loci is sufficiently clear to categorically state that the *AFP* gene is absent in the five other species.

### Phylogenetic comparisons suggest the neighboring genes to AFP were not transferred

Genes flanking the *AFP* gene were evaluated for their usefulness in determining the relatedness of the corresponding smelt and herring sequences. *RBP3-2* and *MMS19* were chosen as they are quite dissimilar to their paralogs. *MMS19* was recovered from the Pacific herring liver and testes transcriptome assemblies [40] but *RBP3-2* was absent as it is only expressed in the eye [42], so it was amplified from Atlantic herring genomic DNA using degenerate primers.

**Figure 5:**
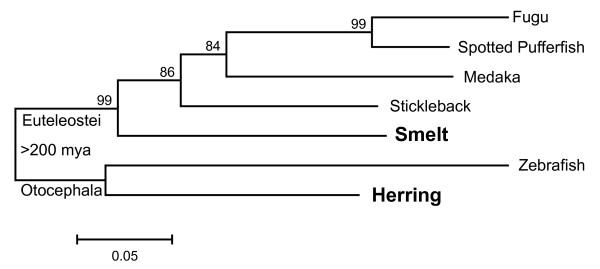
**Maximum-likelihood bootstrap consensus tree derived from an alignment of a portion of the RBP3-2 proteins of various fish.** The names of type II AFP-producing fish are in bold. The scale bar represents 5% divergence. Bootstrap values (500 trials) are indicated at the nodes. The split between Euteleostei and Otocephala occurred over 200 mya [17,18].

Maximum likelihood trees (Figure 5 and Additional file 5: Figure S2C) were constructed from the alignments shown in Additional file 5: Figure S2A, B. These trees are consistent with accepted phylogenies [43-45] and both the smelt and herring sequences lie where expected, within the euteleostean and otocephalan clades, respectively. Thus, these genes arose by descent rather than by LGT.

### The non-coding regions of syntenic genes and the AFP genes differ in their conservation

**Figure 6:**
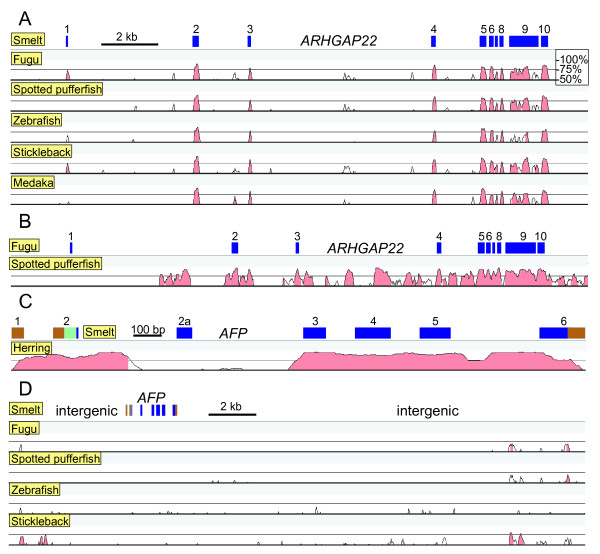
**VISTA comparisons between various fish sequences.** Percent identity is scored over a 100 bp window with a bottom cut-off of 50%. Matches above 70% are colored pink. **A**) Comparison of the *ARHGAP22* orthologs of five fish to that of smelt. Exons in smelt are numbered and indicated by blue rectangles. Ensembl accession numbers for these genes are as follows; fugu (ENSTRUG00000017136), spotted pufferfish (ENSTNIG00000004993), zebrafish (ENSDARG00000076434), stickleback (ENSGACG00000005702), medaka (ENSORLG00000002387). **B**) Comparison as in A but between the sequences of the two pufferfish. **C**) Comparison of the AFP sequences of herring (GenBank DQ003023.1) and smelt (GenBank DQ004949.1). Exons are color coded with brown for non-coding, pale green for signal peptide encoding and blue for mature protein encoding sequence. **D**) Comparison of the intergenic region between the *FRMPD2* and *GDF10* genes using the shuffle-LAGAN alignment. The *AFP* exons are indicated as in C.

Detailed pair-wise sequence comparisons between the conserved syntenic regions of the six fish species, as visualized using VISTA, demonstrated that the intronic sequences have generally not been well conserved within any of the genes. In the example shown (Figure 6A, *ARHGAP22*), the only good alignment to smelt, outside the 10 exons, lies between exons 2 and 3 and likely corresponds to an alternative exon. Only the pufferfish sequences show some similarity within the introns (Figure 6B), as these are the two species of those considered that have diverged most recently (~ 80 million years ago [46]). However, the *AFP* genes of herring and smelt are in stark contrast (Figure 6C) and they show greater overall similarity than even the pufferfish genes. This includes high similarity in the 5′ UTR, four of the five introns, and the 3′ UTR, excepting the insertion in the smelt that is not found in herring. Even here, there is strong similarity across either end of the intron, spanning ~ 200 bases at the 5′ end. Therefore, the conservation of the non-coding sequence is also anomalous, which is again inconsistent with descent but consistent with LGT.

### The intergenic regions flanking the AFP gene are devoid of viral and transposon sequences

Vista comparisons between the 24.5 kb region between the *FRMPD2* and *GDF10* genes in smelt, which contains the *AFP* gene (Figure 1), and the corresponding intergenic regions of the five other fish showed that there are short regions of similarity close to the two flanking genes but no similarities anywhere near the *AFP* gene (Figure 6D). Therefore, there is no evidence for *AFP* pseudogenes in these other fish, which does not support the gene loss hypothesis, but is entirely consistent with LGT.

This intergenic region was split into overlapping segments of 2 kb that were used as queries in both BLASTN (nr and EST databases) and BLASTX (nr database) searches and known transposable element or viral sequences were not detected. The sequence was also scanned using Censor [47] which searches for similarity to repetitive elements catalogued in the repbase database. The 90 putative matches throughout the smelt insert were quite short (<300 bp). Some of these, including the twelve hits within the intergenic regions flanking the *AFP* gene, may represent residual transposon or retrotransposon sequences, but their level of degradation is extensive compared to the *AFP* gene. Therefore, there is no evidence to suggest that this LGT was mediated by a transposon or viral vector.

## Discussion

Lateral gene transfer (LGT) is a well-recognized phenomenon in prokaryotes, particularly where antibiotic resistance is acquired by pathogenic strains of bacteria, conferring a selective advantage that has tremendous consequences for human health [48]. Although LGT is far less frequent in eukaryotes, it has been observed in single-celled eukaryotes [49,50] and the review by Zhaxybayeva and Doolittle provides several salient examples from across the ‘tree’ of life [51]. Despite this, vertical descent is usually taken as the null hypothesis, even in the face of evidence to the contrary, and Keeling and Palmer state that “the erroneous report of substantial HGT in the human genome has probably chilled the field, tarnished more credible claims for eukaryotic HGT” [50]. There may be other examples of lateral *AFP* gene transfer as surprising similarities have been observed between the AFPs of fungi, diatoms and some bacteria, while similar sequences are absent from most other species throughout these three biological domains [52]. Unicellular eukaryotes, including fish parasites [53] and plant parasites [54] have been the recipients of intradomain LGT from other eukaryotes, but given the relative paucity of complete eukaryotic genome sequences, the true extent of such transfers is currently unknown [55].

In higher organisms, for transferred genes to persist, they must be incorporated into the germ line. So, it is not surprising that extensive LGT has occurred between the intracellular bacterial parasite, *Wolbachia*, which infects gonadal tissues, and a number of its host insects and nematodes [56]. Reports of transfers to and between multicellular organisms are on the rise [57], including the previously mentioned transfer of carotenoid biosynthesis genes between fungi and aphids [33]. However, reports of LGT in vertebrates are rare and limited to “selfish” DNAs such as the *SPIN* transposon that has been transferred between 14 families of lizards [58].

There are three main criteria of use in establishing LGT in eukaryotes; unexpected phylogenetic relationships, a patchy phyletic distribution and an overlap of life habits [49], We previously reported the first instance of LGT of an antifreeze gene between two vertebrates. Southern blotting indicated that many intervening species lacked this gene and the sequence of the rainbow smelt AFP differed about as much from that of the Japanese smelt (same family) as it did from the herring (different superorder). We also discussed the possibility of sperm-mediated transformation as a mechanism, whereby sperm absorb foreign DNA fragments, given that these fishes employ external fertilization and have overlapping ranges [16]. A true hybridization, in which an egg was fertilized by the sperm of the other species, is not possible given that herring and smelt belong to different superorders that diverged over 200 million years ago [17-19]. The incompatibility is so severe that even whole nuclei, transplanted into the enucleated cytoplasm of fish eggs from different orders, die early in development [59].

Here we report several new lines of evidence to support the finding of LGT in fishes. A BAC clone, containing the single copy of the *AFP* gene found in a smelt, was sequenced (Figure 1). The corresponding microsyntenic region was identified in the five publicly available genome sequences from fish and was found to be devoid of any *AFP*-like sequences (Figure 4). Not even so much as a pseudogene was found here (Figure 6D), or in any other location within these five genomes. The twelve smelt genes flanking this *AFP* were unremarkable in that they did not appear unexpectedly similar to their homologs in any other species and their intron sequences were unique (Figure 6A). As four of these fish are more closely related to smelt than herring, [17-19] this suggests that the high conservation of the non-coding sequences of the *AFP* between herring and smelt is atypical (Figure 6C) [16]. Phylogenetic gene comparisons indicated that it is only the *AFP* that defies taxonomic conventions (Figure 5, Additional file 5: Figure S2C, [10,16]), suggesting that the *AFP* is the only gene within this region that was laterally transferred.

The recent deposition of numerous EST sequences for Atlantic herring and smelt has allowed us to demonstrate the unusual mutation pattern within the *AFP* gene (Figure 3). All other genes showed similar rates of silent site mutation that were 5- to14-fold higher than that of the *AFP* gene, while the rate of missense mutation in the *AFP* suggests the protein sequence is not under strong selection. This is not likely due to codon selection as several of these other genes also appear to be highly expressed as evidenced by their abundance in the EST database. As well, the codon usage of the *AFPs* does not differ significantly from the other genes.

An unusual feature of the AFP of smelt is that it is encoded by a single-copy gene, whereas the AFPs of herring [16] the AFGPs of numerous notothenioid fishes [60], the type I AFPs of flounders [61] and the type III AFP from ocean pout [26] are all encoded by multigene families in which most of the copies appear to be clustered, sometimes in tandem repeats. The dearth of polymorphisms in the smelt ESTs is consistent with this difference. Gene amplification is a more efficient method of responding to rapid environmental change, as duplication of a gene encoding a weakly active protein is several orders of magnitude more rapid than the acquisition of point mutations that improve activity. It is thought that gene copy number reduction follows once the genes have improved under selection [24], and as this has not yet occurred in other AFP-producing fishes, it would be puzzling for the smelt to be an exception. Smelt is also different than most fish in that it does not rely upon AFP alone for freeze resistance but synthesizes high levels (~0.4 M) of the small molecule antifreeze, glycerol. This drops the serum freezing point close to that of seawater, but this is not the case for herring, which has much lower levels of solutes in wintertime [62,63]. Glycerol is an energetically costly solution to freeze resistance as it is readily lost to the environment, likely through the skin and gills [63]. Although the directionality of the transfer cannot be conclusively determined at this time and the independent evolution of two parallel strategies is not impossible, it seems more plausible that the *AFP* of smelt was acquired secondarily and is an energetically favorable adjunct to an existing antifreeze strategy.

Phylogenetic comparisons indicate that the type II *AFP* gene is more similar to fish lectins (<40% identity) than to those of any other group of organisms [14]. A newly acquired *AFP* gene would confer an immediate selective advantage to fish at risk of freezing in ice-laden waters and as such, it is a likely candidate for LGT since it is not part of a complex of interacting proteins or processes [64]. Since most fish spawn externally in water, their germ line is somewhat vulnerable to DNA uptake. When the evidence is taken together, it all supports the hypothesis that the patchy distribution of highly similar type II *AFP* genes in herring and smelt is a result of LGT between fishes.

## Conclusions

Here we have documented five pieces of new evidence to support the case for the lateral transfer of a lectin-like *AFP* gene between fish species. i) The arrangement of genes in the region where the *AFP* gene is located in smelt is shared by other fishes, but only the smelt has the *AFP* gene. ii) There is also no evidence of *AFP* pseudogenes in these other fish. iii) Genes flanking the smelt *AFP* gene are smelt-like and not herring-like, indicating that the sequenced region is normal except for the *AFP* gene. iv) The introns of genes adjacent to the *AFP* gene are highly divergent between species, which strongly contrasts with the similarity of the introns of the herring and smelt *AFP* genes. v) Lastly, the rate of silent vs non-silent mutation of the *AFP* gene is inconsistent with descent from a common ancestor. As well, smelt is unique in that other species of AFP-producing fishes (including the herring) have multiple copies of their *AFP* genes.

## Methods

### Screening of rainbow smelt BAC library

The rainbow smelt BAC library filters numbers one to three (CHORI-74, [35]) were screened with a 40-mer probe (Table 1) designed from GenBank accession DQ004949. The 40-mer was end-labeled with ^32^PγATP using T4 polynucleotide kinase. Prehybridization, hybridization, imaging and growth of positive clones were performed as in Johnstone *et al*. (2008 [65]). A small amount of DNA from each positive clone was amplified using the 40-mer probe and Type II AFP R primer (Table 1) as previously described [65], except that denaturation was done for 45 s, and the annealing temperature was 65°C.

### Isolation and sequencing of BAC clone

BAC DNA was isolated using the Qiagen® Large-Construct Kit (Qiagen, Mississauga, Ontario) according to the manufacturer’s instructions with the addition of a proteinase K digestion (100 μg/mL) for 1 h at 50°C immediately after the nuclease digestion step, plus two phenol/chloroform extractions and one chloroform extraction once the final pellet was resuspended in 1 ml of 10 mM Tris–HCl, 1 mM EDTA (pH 8). The DNA was then extensively dialyzed against the same buffer. The shearing, shotgun cloning and majority of the sequencing, using an Applied Biosystems 3730xl DNA Analyzer and standard ABI dye-terminator technology, were done by the McGill University and Génome Québec Innovation Centre (Montreal, Canada). Problematic regions were sequenced at Robarts Research Institute (London, Canada) using custom primers flanking the region, in combination with the dGTP BigDye terminator v3.0 kit and/or betaine as needed. The sequence has been deposited under GenBank Accession JQ514278.

### Assembly and gene annotation

The BAC insert sequence was assembled using the CAP3 program [66] and genes identified using overlapping 10 kb segments as the query in a variety of BLAST searches [67] against the non-redundant DNA, protein and EST GenBank databases. Genes were annotated using a wide variety of techniques. This included splice site prediction at the Galaxy website [68] using SplicePredict trained on spotted pufferfish (*Tetraodon nigroviridis*) sequences, comparison with ESTs using GeneSeqer [69] once the ESTs were assembled using the CAP EST Assembler (http://host9.bioinfo3.ifom-ieo-campus.it/cap/), comparisons with protein sequences using Wise2 [70], as well as a variety of gene prediction programs such as Augustus [71].

### Comparisons with other species

The deduced smelt protein sequences were used as queries for BLAST searches on the Ensembl website (http://www.ensembl.org/index.html) [72], against the genomes of other fishes and humans; Zv9 *Danio rerio* (zebrafish) (The *Danio rerio* Sequencing Project (http://www.sanger.ac.uk/Projects/D_rerio/) Wellcome Trust Sanger Institute), BROADS1 *Gasterosteus aculeatus* (stickleback) (The Broad Institute at MIT and Harvard), MEDAKA1 *Oryzias latipes* (medaka) [73], FUGU4 *T. rubripes* (fugu) [74], TETRADON8 *T. nigroviridis* (spotted pufferfish) [75] and human GRCh37 [76]. The corresponding syntenic regions from these species were downloaded and viewed using the Apollo Genome Annotation Tool v1.11.6 [77]. Genes were reannotated, when necessary, using the techniques mentioned above. mVISTA [78] was used for between species comparisons using LAGAN alignments, unless otherwise mentioned.

### Determination of d_N_/d_S_ ratios

All available Atlantic herring sequences were downloaded from the non-redundant and EST databases and mitochondrial sequences, short sequences as well as non-protein coding sequences, such as satellite DNA and rDNA were removed. Following contig assembly using DNAMAN v6 (Lynnon Corporation, Pointe-Claire, Canada) and the CAP3 program [66], homologous smelt sequences were identified using a TBLASTX search. These were categorized as orthologs if both the smelt and herring sequence showed the highest similarity to the same stickleback or zebrafish sequence using TBLASTN and/or BLASTP (http://www.ensembl.org/index.html). Codon usage and percent identity were determined using DNAMAN. Codon aligned orthologous sequence pairs were generated by removing gaps, ambiguous regions including runs of single amino acids with gaps, misidentified introns or likely sequencing errors within poorly represented ESTs. Only alignments longer than 150 bp in length were used to calculate the ratio of non-synonymous changes per non-synonymous site to synonymous substitutions per synonymous site (*d*_*N*_*/d*_*S*_) using SNAP (Synonymous Non-synonymous Analysis Program) [79].

### Phylogenetic comparisons

Portions of the Altantic herring gene encoding the *RBP-3*-2 sequence were amplified from genomic DNA by PCR as above but with an annealing temperature of 50°C. Two fully degenerate primers (Table 1, named inner), corresponding to amino acids 414–421 and 442–449 of zebrafish NP_571526.2, were used to obtain a 61 bp segment. Specific primers were then designed and used in combination with degenerate primers corresponding to amino acids 235–242 and 472–479 to obtain two overlapping segments spanning three exons. Fragments were cloned using the TOPO® PCR Cloning Kit (Life Technologies, Burlington, Canada) and sequenced at Robarts Research Institute (London, Canada). *MMS19* sequences were obtained as described above, except for the Pacific herring sequence that was deduced from the transcriptome data [40]. Phylogenetic analysis of the aligned sequences was done using MEGA 5.01 [80]. The WAG + I and JTT + G substitution models were the best fit for the RBP-3-2 and MMS-19 alignments respectively. Maximum likelihood analysis with gap deletion was performed using 500 bootstrap replicates.

### Quantitative PCR (qPCR)

qPCR reactions were performed with forward and reverse primers for *AFP*, *LDH-A*, *LUC7La* and pBAC-GMR sequences using both smelt genomic DNA and BAC DNA as templates. The primer sequences are listed in Table 1. Five serial dilutions of the DNA were used for each qPCR reaction. The qPCR assay mixture comprised: 12.5 μL of Quanta SYBR, 2 μL of 5 μM forward primer, 2 μL of 5 μM reverse primer and 7.5 μL of nuclease-free water per reaction. The qPCR protocol was: 95°C for 3 min, followed by 40 cycles of 95°C for 15 s, 65°C for 30 s, and 72°C for 15 s. The reactions were recorded and analyzed by the program RQ manager 1.2.1. The efficiencies of qPCR reactions for each set of primers were calculated as: E = (10^(−1/slope)^-1) × 100 where the slope is derived from the linear regression line of the plot log (DNA concentration) vs. average CT of the 5 serial dilutions for each set of primers using either BAC DNA or genomic DNA as template.

## Abbreviations

AFP(s): Antifreeze protein(s); AFGPs: Antifreeze glycoproteins; BAC: Bacterial artificial chromosome; (*d*_*N*_*/d*_*S*_): Ratio of non-synonymous changes per non-synonymous site to synonymous substitutions per synonymous site; LGT: Lateral gene transfer; qPCR: Quantitative PCR; TH: Thermal hysteresis; C-type lectin: Calcium-dependent type lectin.

## Competing interests

One of the four authors (PLD) is a joint holder of a patent on a process of microinjection of DNA into salmonid eggs that can be used to make transgenic fish. PLD also holds shares in a company (AquaBounty) http://www.aquabounty.com/ that is attempting to market transgenic salmon as food.

## Authors’ contributions

JL and WSD isolated the BAC clones containing the *AFP* gene and measured the gene’s copy number. Compilation, analysis and annotation of the smelt AFP locus and syntenic regions in comparative species, and other experimentation, were performed by LAG with input from PLD. The manuscript was written by LAG with editorial advice from JL, WSD, and PLD. All authors read and approved the final version.

## Supplementary Material

Additional file 1 **Figure S1.** Comparisons of BAC clones. Click here for file

Additional file 2 **Table S1.** List of BAC restriction fragments. Click here for file

Additional file 3 **Table S2.** qPCR Ct values for various loci in the BAC and genome. Click here for file

Additional file 4 The pattern and rate of mutation in the smelt *AFP* gene and its absence in the syntenic regions of other fishes is inconsistent with vertical descent. Click here for file

Additional file 5 **Figure S2.** Alignment of RBP3-2 protein sequences. Click here for file
