# Supplementary material for: Smelt was the likely beneficiary of an antifreeze gene laterally transferred between fishes
Source: BMC Evol Biol. 2012 Sep 25;12:190. doi: 10.1186/1471-2148-12-190 (PMC3499448; doi:10.1186/1471-2148-12-190)
Supplement: Additional file 1 — Figure S1. Comparisons of BAC clones. [file 1471-2148-12-190-S1.pdf]

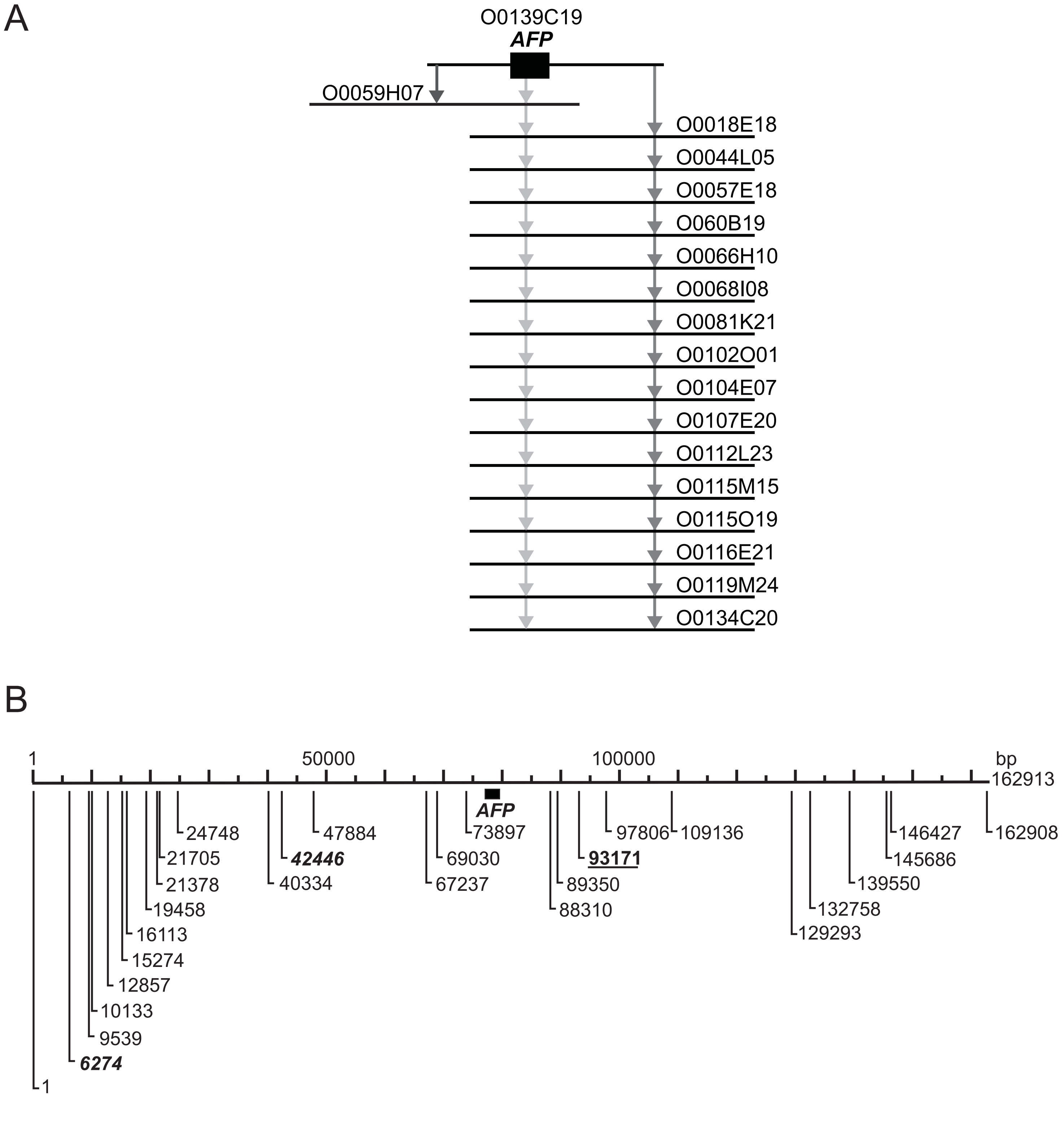

Supplementary Figure S1. Comparisons of BAC clones containing the *AFP* gene. A) Minimum tiling path for 18 rainbow smelt BACs that contain the *AFP* gene. The arrows indicate PCR positive amplifications using primers designed from either end of the sequenced BAC (O0139C19) or the *AFP* gene. B) Location of *Eco*RI sites in the sequenced BAC insert, O0139C19. Restriction sites corresponding to the 5′ ends of O0119M24 and O0068I08 are in bold italics and the 3′ end of clone O0059H07 that overlaps the 5′ end is underlined in bold. The location of the *AFP* gene is indicated with a solid bar.
