# Supplementary material for: Smelt was the likely beneficiary of an antifreeze gene laterally transferred between fishes
Source: BMC Evol Biol. 2012 Sep 25;12:190. doi: 10.1186/1471-2148-12-190 (PMC3499448; doi:10.1186/1471-2148-12-190)
Supplement: Additional file 2 — Table S1. List of BAC restriction fragments. [file 1471-2148-12-190-S2.pdf]

Table S1: Fragment sizes observed upon digestion of three BAC clones with *Bgl*II compared to the expected sizes based upon the sequence of O0139C19.

Coordinates[1]	Genes[2]	Size	Bands observed in BACs[3]		
			O0139C19	O0119M24	O0068I08
694-958	***LUC7La***	265	nd		
959-5628	***LUC7La***	4670	✔	X	X
5629-16552	←***EPS8L1a***	10924	✔	L	X
16553-21446	*LRRC18*	4894	✔ T	✔ D	x
21447-26546	←*ARHGAP22*	5100	✔	✔	x
26547-32339	*ARHGAP22*	5793	✔	✔	X
32340-33921	*ARHGAP22*	1582	✔	✔	X
33922-37110	*ARHGAP22*	3189	✔ D	✔ D	x
37111-38170	*ARHGAP22*	1060	✔	✔	x
38171-42643		4473	✔ D	✔ D	x
42644-46909	*MAPK8*	4266	✔	✔	✔
46910-69170	*MAPK8*	22261	✔	✔	L
69171-72375	←*FRMPD2*	3205	✔ D	✔ D	✔
72376-75797		3422	✔ D	✔	✔
75798-80241	*Type II AFP*	4444	✔ D	✔ D	✔
80242-80253		12	nd	nd	nd
80254-81226		973	✔	✔	✔
81227-88949		7723	✔	✔	✔
88950-89379		430	nd	nd	nd
89380-93089		3710	✔	✔	✔
93090-102369	*GDF10*	9280	✔	✔	✔
102370-105884	←*GDF2*	3515	✔	✔	✔
105885-110726	←*RBP3-1 & -2*	4842	✔ T	✔ D	✔
110727-119282	←*RBP3-2*	8556	✔	✔	✔
119283-134962	←*ANTXR1a*	15680	✔	✔ D	✔
134963-141404		6442	✔	✔	✔
141405-143868		2464	✔	✔	✔
143869-145646	*UBTD1*	1778	✔	✔	✔
145647-148018	*UBTD1*	2372	✔	✔	✔
148019-154307	*UBTD1*	6289	✔	✔	✔
154308-154863	←*MMS19*	556	nd	nd	nd
154864-161672	*MMS19*	6809	✔	✔	✔
161673-165116	*ZDHHC16*	3444	✔ D	x	x
165117-167183	←BAC	2067	✔	✔	✔
167184-693	BAC+*LUC7La*	4930	✔ T	x	x
Unique bands				1	5-6

[1] First base of insert = 1.
[2] Arrows indicate that the gene or vector is partially contained in the preceding fragment.
[3] D or T = intensity consistent with a doublet or triplet, nd = expected but not detected, L = possible match but fragment too large to size accurately, X = clearly absent, x = appears to be absent based on reduced intensity but overlapping bands are present.
