# Supplementary material for: Smelt was the likely beneficiary of an antifreeze gene laterally transferred between fishes
Source: BMC Evol Biol. 2012 Sep 25;12:190. doi: 10.1186/1471-2148-12-190 (PMC3499448; doi:10.1186/1471-2148-12-190)
Supplement: Additional file 3 — Table S2. qPCR Ct values for various loci in the BAC and genome. [file 1471-2148-12-190-S3.pdf]

Table S2. Average of triplicate CT values obtained by qPCR for each primer set using either BAC O0139C19 DNA or genomic DNA as the template

Log (ng/uL)		average CT (*AFP*)	average CT (*LUC7La*)
BAC DNA	average CT (vector)		
2.274	20.00	19.81	20.27
1.973	20.93	20.70	21.09
1.672	22.02	21.70	22.05
1.371	22.93	22.66	23.10
1.070	24.00	23.92	24.09
Genomic DNA	average CT (*LDH-A*)		
2.079	19.95	19.87	19.94
1.778	21.05	20.87	20.92
1.477	22.03	21.87	21.93
1.176	23.04	22.88	22.96
0.875	24.01	23.89	23.96
