# Supplementary material for: Smelt was the likely beneficiary of an antifreeze gene laterally transferred between fishes
Source: BMC Evol Biol. 2012 Sep 25;12:190. doi: 10.1186/1471-2148-12-190 (PMC3499448; doi:10.1186/1471-2148-12-190)
Supplement: Additional file 4 — The pattern and rate of mutation in the smelt AFP gene and its absence in the syntenic regions of other fishes is inconsistent with vertical descent. [file 1471-2148-12-190-S4.pdf]

Supplementary material

The pattern and rate of mutation in the smelt AFP gene is inconsistent with vertical descent

Gene sequences drift at different rates, both between and within genes, depending upon a number of factors including the type and strength of natural selection. Our previous comparison of the *AFP* and the highly-conserved *PRP8P* gene sequences in herring and smelt indicated that the rate of mutation at different types of sites within the *AFP* gene was unusual [1]. Most single base substitutions within coding sequences fall into two categories, the first of which are missense (non-synonymous) mutations which alter the amino acid that is encoded. The second are silent (synonymous) changes that generally occur in the third position of the codon and do not alter the encoded amino acid. Since codons range from a degeneracy of one to six, and multiple substitutions can occur within a single codon, these rates are estimated and normalized [2]. The resulting d_N/d_S ratio is a measure of the rate of non-synonymous changes per non-synonymous site (d_N) over the rate of synonymous change per synonymous site (d_S). When proteins are under strong selection, the rate of missense substitution is generally low, but the rate of silent substitution is not similarly affected, so the d_N/d_S ratio is much less than one. If selection is relaxed, the ratio can be much closer to one. We previously determined that the d_N/d_S ratio for the mature coding sequence of the *AFP* was close to unity, whereas that of the *PRP8P* gene was very small [1]. This naively suggested that there was much weaker selection on the AFP sequence, which is clearly inconsistent with its higher conservation at the nucleotide level.

The recent deposition of additional sequences, such as 397 herring liver ESTs [3], facilitated an extension of this previous analysis. The herring sequences were assembled into a total of 93 contigs which were used to identify smelt homologs. These were categorized as orthologs if the herring and smelt sequence were most similar to the same sequence from stickleback (*Gasterosteus aculeatus*) and/or zebrafish (*Danio rerio*), and once two were excluded for being too short, thirteen remained. Several paralogs from large gene families (parvalbumin, actin and myosin) were also analyzed. The d_N/d_S ratios of these genes were compared to their overall sequence identity at the protein level (Figure 3 of the manuscript). This ratio is low (< 0.2) in proteins that are well conserved, in contrast to the *AFP*, which has a ratio of 0.64. Even proteins under reduced selection, with identities below 50%, have ratios that are less than half that of the *AFP*. As protein sequence identity increased, the ratio of non-synonymous differences per non-synonymous site (d_N) was of course lower, but the corresponding ratio for synonymous differences (d_S) did not change dramatically (~3-fold range). The exception is the *AFP* gene; in which d_S was 5- to 14-fold lower than in the other genes. This is reflected in the sequence identities. There are two proteins with higher identity (99.6% and 92%) than the AFPs (85%), but it is the *AFP* that has the highest overall DNA sequence identity (92% vs. 89% and 87%, respectively). The paralogs, encoding the lower identity parvalbumins, myosin and a highly similar actin showed the same trend as the orthologous pairs with ratios distinctly different than the *AFP*. This analysis indicates that the conservation between the coding sequences of the smelt and herring *AFP* is atypical compared to other genes, with much lower rates of synonymous mutation. This result is inconsistent with homology by descent but consistent with what would be expected for LGT.

A detailed analysis of the syntenic regions of other fishes shows the absence of the AFP gene

The general characteristics of the proteins encoded by the fourteen genes flanking the smelt *AFP* gene were deduced by homology and their arrangement is shown in Figure 1 of the manuscript. LUC7La is a spliceosomal protein, LRRC18 contains leucine-rich repeats, EPS8L1a may be involved in signaling and ARHGAP22 is a RhoGTPase activating protein. FRMPD2 contains FERM and PTZ domains which may mediate binding to the membrane and other proteins. *FRMPD2* was difficult to annotate as it may have undergone a partial duplication, giving rise to "orphan" exons (*FRMPD2-like*). MAPK8 is a protein kinase; GDF10 and GDF2 are growth factors; RPB3-1 and RBP3-2 are both highly similar to RBP3 (retinol-binding protein 3), ANTXR1a is involved in cell attachment and migration; UBTD1 contains a ubiquitin-like domain; MMS19 is an excision repair homolog; and ZDHHC16 contains a zinc finger and is likely a palmitoyltransferase.

The corresponding syntenic regions from five other fishes with sequenced genomes were compared to that of the smelt, primarily to ascertain whether *AFP* gene sequences are present. Secondary information, such as differences in gene arrangement and the relative conservation of sequences within the coding and non-coding regions of the genes were also ascertained. These data are presented in Figures 4 and 6 of the manuscript.

The most complete and best annotated corresponding syntenic region was from the 'fugu' pufferfish, *Takifugu rubripes* (Ensembl accession

FUGU4:scaffold_3:3093000:3265000:-1) [4]. It contains only one gap, found within an intron of the *ARHGAP22* gene (3rd from the left). The first two genes found on the smelt BAC insert, *LUC7La* and *ESP8*, are located about 1.8 MB away, on the same chromosome. This difference represents a genuine rearrangement in smelt as a second BAC insert (O0119M24), which was end sequenced, starts within the third intron of the fourteen-exon-long *ESP8* gene. In fugu, a gene of unknown function that contains an immunoglobulin-like domain (*VSTM4*) is found in their place, as it is in humans ([5] not shown). All of the remaining genes, except the *AFP* gene, are found in the same order as in smelt. The region corresponding to the *AFP* gene was devoid of functional genes in fugu. There is a region that encodes a sequence highly similar to translation initiation factor eIF4A II. However, this copy does not contain any of the introns normally associated with the *eIF4A II* gene and it also contains a frameshift mutation, suggesting that this is merely a non-functional retrocopy derived from a mature mRNA transcript. This retrocopy is not found in smelt or any of the other species, suggesting its insertion was a relatively recent event.

The sequence from the green spotted pufferfish, *Tetraodon nigroviridis* (Ensembl accession TETRAODON8:2:5230000:5400000:1) [6], shows the same arrangement of genes as fugu. However, there are twenty gaps with size estimates ranging from 20 bp to 3 kb, several of which are clustered near the 5′ end, preventing determination of whether *VSTM4* or *ESP8-like* is found upstream of *LRRC18*. As well, two of the genes contain frameshift mutations that significantly alter the open-reading frame. These may represent sequencing errors as they are not found in the corresponding cDNA sequences. There is but a single copy of the *RBP3* gene in this region. However, unlike the single copy in

humans (not shown), this gene contains two rather than four copies of the peptidase S41 domain. Again, there are no apparent genes found in the region corresponding to the *AFP* gene in smelt.

The equivalent sequence from the zebrafish, *D. rerio*, was found near the end of chromosome 12 (Ensembl accession Zv9:12:2660000:3450000:1) (The *Danio rerio* Sequencing Project (http://www.sanger.ac.uk/Projects/D_rerio/) Wellcome Trust Sanger Institute). The gene arrangement is identical to that found in fugu, including the absence of *LUC7La* and *ESP8* and the presence of *VSTM4*. However, this segment contains nine gaps of unspecified length, five of which are found within the region between *FRMPD2* and *GDF10*, the two genes flanking the *AFP* gene in smelt. This region is also quite large (~0.37 Mb) and contains a number of putative genes (not shown), although none encode an AFP or C-type lectin. Most of these predicted genes are highly dubious and some overlap repetitive DNA, such as the one that predicts an exon in each of 28 slightly variable copies of a 1291 bp sequence that is also found in clusters on chromosome 4. In general, the length of the introns and other intervening sequences average over twice those of the other species. This assembly may not yet be accurate as the previous build found the *FRMPD2* and *GDF10* genes much further apart and on opposite strands. The names assigned to the fish genes are often based on their similarity to human genes, but they do not necessarily match those given here. For example, *FRMPD2*, which is a member of a multi-gene family, was named *tyrosine-protein phosphatase non-receptor type 13 isoform 3* in zebrafish, after the human paralog, *PTPN13*.

The corresponding syntenic region of the medaka, *Oryzias latipes* (Ensembl accessionMEDAKA1:19:210000:300000:-1 and MEDAKA1:ultracontig222:1:115000:1)

[7], has similar problems to the zebrafish sequence. However, in this case, the left-hand portion of the region is found on chromosome 19 whereas the right-hand portion is not yet positioned. Nevertheless, nothing resembling the *AFP* gene is found flanking either the *FRMPD2* or *GDF10* genes.

Lastly, the corresponding syntenic region of the stickleback, *G. aculeatus*, shows the same gene order as the other species, from *VSTM4* to *GDF10* (Ensembl accession BROADS1:groupV:7540000:7717500:1) (The Broad Institute at MIT and Harvard). However, gaps become an issue in the vicinity of *GDF10*, such that an exon is absent. These gaps also make it unclear as to the accuracy of the assembly downstream of this gene. Again, nothing resembling an *AFP* gene is found here.

References

1. Graham LA, Lougheed SC, Ewart KV, Davies PL: **Lateral transfer of a lectin-like antifreeze protein gene in fishes**. *PLoS ONE* 2008, **3**(7):e2616.
2. Nei M, Gojobori T: **Simple methods for estimating the numbers of synonymous and nonsynonymous nucleotide substitutions**. *Mol Biol Evol* 1986, **3**(5):418-426.
3. Olsvik PA, Waagbo R, Pedersen SA, Meier S: **Transcriptional effects of dietary exposure of oil-contaminated Calanus finmarchicus in Atlantic herring (Clupea harengus)**. *J Toxicol Environ Health A* 2011, **74**(7-9):508-528.
4. Aparicio S, Chapman J, Stupka E, Putnam N, Chia JM, Dehal P, Christoffels A, Rash S, Hoon S, Smit A *et al*: **Whole-genome shotgun assembly and analysis of the genome of Fugu rubripes**. *Science* 2002, **297**(5585):1301-1310.
5. Consortium IHGS: **Finishing the euchromatic sequence of the human genome**. *Nature* 2004, **431**(7011):931-945.
6. Jaillon O, Aury JM, Brunet F, Petit JL, Stange-Thomann N, Mauceli E, Bouneau L, Fischer C, Ozouf-Costaz C, Bernot A *et al*: **Genome duplication in the teleost fish Tetraodon nigroviridis reveals the early vertebrate proto-karyotype**. *Nature* 2004, **431**(7011):946-957.
7. Kasahara M, Naruse K, Sasaki S, Nakatani Y, Qu W, Ahsan B, Yamada T, Nagayasu Y, Doi K, Kasai Y *et al*: **The medaka draft genome and insights into vertebrate genome evolution**. *Nature* 2007, **447**(7145):714-719.
