# Supplementary material for: Smelt was the likely beneficiary of an antifreeze gene laterally transferred between fishes
Source: BMC Evol Biol. 2012 Sep 25;12:190. doi: 10.1186/1471-2148-12-190 (PMC3499448; doi:10.1186/1471-2148-12-190)

Supplementary Figure S2. Phylogenetic comparisons of the deduced protein sequences of two genes from various fishes from the microsyntenic region containing the *AFP* gene in smelt. A) Alignment of a portion of the RBP3-2 sequences. Residue conservation is indicated by asterisks for identity, colons for high similarity and periods for low similarity. The herring sequence was amplified from Atlantic herring (*Clupea harengus*) DNA as described in the main manuscript. When the sequences in the Ensembl genomes were inconsistent with conspecific ESTs and/or the sequences from other fishes or were not annotated, the genes were either reannotated or the assembled EST sequences from NCBI were used. The Ensembl (EN...) NCBI or NCBI UniGene accession numbers are as follows; fugu (*Takifugu rubripes*) ENSTRUP00000043868 (reannotated), spotted pufferfish (*Tetraodon nigroviridis*) ENSTNIP00000007356, medaka (*Oryzias latipes*) ENSORLP00000025163, stickleback (*Gasterosteus aculeatus*) Gac.8266, smelt (*Osmerus mordax*) JQ514278, zebrafish (*Danio rerio*) ENSDARP00000118871 . B) Alignment of a portion of the MMS19 sequences as above. The accession numbers are as follows; fugu ENSTRUP00000043827 (reannotated) spotted pufferfish ENSTNIP00000007353 (reannotated), medaka ENSORLP00000025202 (reannotated), stickleback (reannotated based upon DW644435.1), zebrafish ENSDARP00000098600 (reannotated based upon numerous ESTs). The herring sequence was obtained from the Pacific herring (*Clupea pallasi*) as described in the main manuscript and missing sequence is indicated with question marks whereas gaps are indicated with dashes. C) Maximum-likelihood bootstrap consensus tree derived from an alignment of the MMS19 proteins from above. The names of type II AFP-producing fish are in bold. The scale bar represents 5% divergence. Bootstrap values (500 trials) are indicated at the nodes. Regions containing gaps or missing sequence were excluded from the analysis. The frog (*Xenopus laevis*) sequence (GenBank NP_001087156.1) was used as the outgroup for MMS19 but this was not done for RBP3-2 as the protein contains repeats of the peptidase S41 IRBP domain (GenBank cd07563) that are variably amplified in fishes and non-fishes, making an accurate alignment difficult.

A

```
Fugu        LKNLKRATIVGERTAGGSVKLDNFKVGSTDFYITVPTAKSINPVTGSSWEITGVKPDVEV 60
Spotted     LKNLKRATIVGEKTAGGSLKLDTFKVGDTDFYITVPTAKSINPITGSSWEIRGVTPHVEV 60
Medaka      LKNLKRATIVGEKTAGGSAKIKKFRVGDTDFYVTLPTAKSINPITGSSWEVTGVKPNVEV 60
Stickleback LQNLKRATIVGEKTAGGSVKVDKIQVRDTGFYVTVPTAKSVNPITGSTWEVTGVTPNVEV 60
Smelt       LKNLKRATVVGEKTAGGSVKIDKIKVGDSDFYLAIPSAKSINPITGNTWEVTGVTPDIEV 60
Herring     LQSLKRATLVGEHTAGGSVQIDKMEVGDTDFYVSVPVAKSTNPVTGKTWEVVGVTPDVEV 60
Zebrafish   LKNLKRATIVGENTAGGTVKMSKMKVGDTDFYVTVPVAKSINPITGKSWEINGVAPDVDV 60
            *:.*****:***.****: ::..:.* .:.**:::* *** **:**.:**: ** *.::*

Fugu        NAEDALATAIKIVSLRAQIPAIIEGAATLIAKNYAFEATGADVATKLRELLAKGQYNSVV 120
Spotted     NAEDALATAIKIVNLRAQIPAIIEGTAALVANNYAFEATGADVAKELRELQANGQYSSVV 120
Medaka      NAEEALATALKIINLRLQVPAIIEESATLVANNYAFESTAADVAEKLKGHLANGDYNMVV 120
Stickleback NAEDALATAIKIVTLLNQVPAIIEGSATLIADNYAFEDIGAAVAEKLKGLLANGEYSKVV 120
Smelt       DPEDALAKAIEIINLRAQIPAIVEGAGALVADNYAFQSVGADVAEKLNGILASGDYSMVS 120
Herring     AAEDALDVALRIINLRAQIPELLQASGALVAENYAFESVGADVSEKLAALAASGEYDMIN 120
Zebrafish   AAEDALDAAIAIIKLRAEIPALAQAAATLIADNYAFPSIGEHVAEKLEAVVAGGEYNLIS 120
             .*:**  *: *:.*  ::* : : :.:*:*.****   .  *: :*    * *:*. :

Fugu        SSESLEVALSADLQRLSGDKSLKATQNAPVLPPMDYSPEMYIELIKVSFHTDVFENNIGY 180
Spotted     SKESLEAALSADLQRLSGDKSLKTTPNTPVLPPMDYTPEMYIELIKVSFHTDVFENNIGY 180
Medaka      SKESLEAKLSADLQSLSGDKSLTVSSNTGAPPPMEYTPEMYIELIKISFHTDVFENNIGY 180
Stickleback SKDSLEMKLSADLRTLSGDKSLKTTSNVPALPPMNYSPEMYIELIKVSFHTDVFEDNIGY 180
Smelt       SKEELEIKLTADLKTLSGDKSLRTTHNTPVLPPMDYTPEMFIQLIKVSFQTDVFENNIGY 180
Herring     SKEDLEVKLSADPQKLSGDKSLTTTSNTPALPPMNPTPEMFIELIKVSFHTDILENNIGY 180
Zebrafish   TKEDLEERLSEDLLKLSEDKCLKTTSNIPALPPMNPTPEMFIALIKSSFQTDVFENNIGY 180
            :.:.**  *: *   ** **.* .: *  . ***: :***:* *** **:**::*:****
```

```
Fugu        LRFDMFGDFEEVKAIAQIIVEHVWNKVVNTDALILDLRNNVGGPTTAIA 229
Spotted     LRFDMFGDFEEVKAIAQIIVEHVWNKVVNTDALILDLRNNVGGPTTAIA 229
Medaka      LRFDMFGDFEEVKAIAQVIVEHVWNKVLHTDAMIIDLRNNVGGPTTAIA 229
Stickleback LRFDMFGDFEEVKAIAQIIVEHVWNKVVNTDAMIVDLRNNIGGPTTAIA 229
Smelt       LRFDMFGDFEEVRAIAQIIVEHVWNKVVNTDGMIVDLRNNIGGPTTAIA 229
Herring     LRFDMFGDFEQVKAIAQVVVEHVWNKVVDTDALIVDLRNNVGGPTTSIA 229
Zebrafish   LRFDMFGDFEHVATIAQIIVEHVWNKVVDTDALIIDLRNNIGGHASSIA 229
            **********.* :***::********:.**.:*:*****:** :::**
```

B

```
Fugu        MAADSSLLPSLVEEYVSGLQDSKAKDTATGVKGGQFTIVQLVEALGLSLTSSQPHTRARG 60
Spotted     MAADAPSLPSLVDEYVSGLQDSKAKDTATGVKEGQFTILQLVEALSQSLTSSQPPTRARG 60
Medaka      MAADGALLLSLVEEFVSGLQDSKAKDAAKGVKDGAFTVLQLVEALGPSLTSSQPQTRARG 60
Stickleback MAADSAVLLSLVEEFVSGLQDSKANDAATCVKDGQFTILELVEALGLSLTSSQPHTRARA 60
Smelt       MAADSALLVGLVEEFVSGQQDSKAADTATGIKAGKFTILQLVEALGLSLTSSQPQTRARA 60
Herring     ??????????????????????????????????????????????????????????RG 2
Zebrafish   MAADNNVLLGLVEEFVSGQVDSKAADTSTGVKNGQFTVLQLVEALGVSLTSSQPQTRGRG 60
Frog        MAEAQTALWGLVEEFVVGEQDSKSAEVAAGVKDGVFTVLQVVESLGSCLANPEPRMRSKG 60
                                                                      :.

Fugu        VQLLSEVLQECYGVFTVAEVEVLISFYENRLKDHHVVIPPVLQGLRALTKCTVLPPGSAV 120
Spotted     VRLLSDVLGECYAVFTEAELKVLITFYENRLKDHYVVIPPVLQGLRALTKCRVLPPGSAV 120
Medaka      VQLLSEVLQDGRAELSQKEVEVLMAFYQNRLKDHHVTTPPVLRGLLALTKCAALPPGSAV 120
Stickleback VQLLSQVLHDCHGALTEREVEVLLAFYENRLKDHYVITPPVLHGLRALTKCTVLPPGSAV 120
Smelt       VQLLSQVLQECYADLSEQEVEVLIAFYENRLKDHYVITPHALQGLKALTKCTVLPPGSAV 120
Herring     VKLLSQVLQECYSYLNEREVEVLVAFYENRLKDHYVIIPPVLQGLKALTKSSALPPGSAV 62
Zebrafish   VQLLSQVLQECYSGLSEREVEVLIAFYENRLKDHYVITPHVLRGLKALAKCSVLPPGSAV 120
Frog        VQLLSRVLLECYSRLTEKEVEVLVVFYENRLKDHHLITPHVLQGLMALSMCDVLPQGVAV 120
            *:*** ** :  . :.  *::**: **:******::  * .*:** **: . .** * **

Fugu        AMLRALFQDVHVQSLMLAERACVYNMLLNLMETREAELKGLGADFVFGFVQSMDGERDPR 180
Spotted     AMLRAVFQDVHVQSLMLAERACVYSMLLNLMETREAELKGLGADFVFGFVQSMDGERDPR 180
Medaka      SMLRSLFQDVHVQSLMLAERACVYNMLTNLMETREAELKSLGADFVFGFVQSMDGERDPR 180
Stickleback SMLRSLFQDVHVQSLMLGERACVYNMLINLMATREAELKGLGADFVFGFVQSMDGERDPR 180
Smelt       SMLKSLFQDVHVQSLMLAERAHVYNMLINLMGSREEELKGLGADFVFGFVQSMDGERDPR 180
Herring     SILKSVFQDTHVQSLMIQERSCVYNILMNLMESREQELKGLGSDFVFGFVQSMDGERDPR 122
Zebrafish   SILKSIFQDVHVQSLMVTERSCVYNILISLMESREEELKGLGADFIFGFVQSVDGERDPR 180
Frog        SVLKSVFQEVHVQSLMQIDRHTVYMIITNFMKTREEELKNLGADFTYGFIQVMDGEKDPR 180
            ::*:::**:.******  :*  ** :: .:* :** ***.**:** :**:* :***:***

Fugu        NLLLAFQVAKNIIHRGYELGSFTEEMFEVTSCYFPIDFAPPPNDPHGITREELVVTLRDV 240
Spotted     NLLLAFQVAKNIIQRGYELGGFTEEMFEVTSCYFPIDFTPPPNDPHGITKEQLVVTLRDV 240
Medaka      NLLLAFRIARNLIVQGYDLGKFAEELFEVTSCYFPIDFTPPPNDPHGITKEELVQALRAV 240
Stickleback NLLLAFQIARTIVLRGYDLGKFTEELFEVTSCYFPIDFSPPPNDPHGVTREELIQMLRDV 240
Smelt       NLLLAFQIARNIIHRNYDMGKFTEELFEVTSCYFPIDFSPPPNDPHGITKEELILTLRAV 240
Herring     NLLLSFQIAGNIIAHGYEMGKFTEELFEVTSCYFPIDFTPPPNDPHGITQEDLILSLRAV 182
Zebrafish   NLLLAFQVAKNIIYRGYDLGKFVEELFEVTSCYFPIDFSPPPNDPHGITQEELILSLRAV 240
Frog        NLLVAFYIVQDIVTKNYALGPFVEELFEVTSCYFPIDFTPPPSDPHGITREHLIMGLRAV 240
            ***::* :.  :: :.* :* *.**:************:***.****:*:*.*:  ** *
```

```
Fugu        LVGTPKFAEFLLPLIIEKLDSDVQSAKLDSLQTLTACVTRYERKDLAEFLGGLWASLRRE 300
Spotted     LVGTPKFAEFLLPLIIEKLDSDVQSAKLDSLQTLTACVSQYEHKDLAEFLEGLWASLRRE 300
Medaka      LTGTPKFAEFLFPLLIEKLDSEVQSAKLDALQTLNACLSRFEHKDTAEFLQGLWSSLRRE 300
Stickleback LTGTPRFAEFLLPLIIEKLDSDVQSAKLDSLQTLVAGVSKYEHKDVAEFLEGLWASLRRE 300
Smelt       LTGTPSFAEFLLPLIIEKLDSDVQSAKLDSLQTLSACASVYEYKDLAEFLPGLWASLRRE 300
Herring     LTGTPLFAEFLMPLIIEKLDSDVQSAKVDSLQTLAACGPVYGHKELAQFLSGLWSSLRRE 242
Zebrafish   LTGTPRFAEFLLPLIIEKMDSDVQSAKVDSMHTLAACGQTYSHKELAEFLPGLWSSIRRE 300
Frog        LASTSRFAEFLLPLLIEKMDSEMQSAKLDSLQTLSACCTVYGQKELKEFLSGLWSSIRRE 300
            *..*. *****:**:***:**::****:*:::** *    :  *:  :** ***:*:***

Fugu        VFQTASEKIESASLAAVTALTSCLSRSVLKSDSEDSLHSFLELVLKGCKHHLCEPDLKLV 360
Spotted     VFQTASEKIESASLAAVTALASCLSRSVLKSDSEDSLRSFLDLVLRDCRHHLSEPDLKLV 360
Medaka      VFQTSSEKIESAALAALAALTSCLSRSVLSSESEDVLGRFLEVVLRDCEHHLREPDLKLV 360
Stickleback VFQTASEKIEAAGLAALTALTSCLSRSVLNSDSEDSLSTFLQLVLTDCKHHLCEPDLKLV 360
Smelt       VFQTASERVESTGLSALSALTSCLSRSVLNSDSEDSLNVFLDQVIKDCEHHLSEPDLKLV 360
Herring     ????????????????????????????????????????????????????????????
Zebrafish   VFQTASERVESAGLSALSSLVSCLSRSVLNSDSEDSLQVFLNLVLKDCQHHLCEPDLKLV 360
Frog        VFQTASEKIEAEGLAALQALSACLSRSVLSPDAEDLLDSFLNNILQDCKHHLCEPDMKLV 360


Fugu        WPSAKLLQAACSASNRAGHIITSAVMPLLTEQYHSRTECSHRRTLLEVVQKFIQAVKPRQ 420
Spotted     WPSAKLLQAACGGSNRAAHTITAAVMPLLSEQYHSRTQCSHRRTLLEVVQKFIESVKLCQ 420
Medaka      WPAAKLLQACCGASYRASRVITAAVLPSLTEQYNGKTQCSQRRTLLEVLQRLVQSVKSSP 420
Stickleback WPSAKLLQAACAASSRASHTVADAVMPALIEQYNGRAQCSQRRTLLEVVQRFLQSVKTCE 420
Smelt       WPSAKLLQAASSASYRASYRVAAAVMPSLIEQYNNRTQCAHRRTLLEVVQGFVQPSKIGQ 420
Herring     ??????????????????????????PLLLEQYINRTQSAQRRTLLEVLQGFIQPTEASR 276
Zebrafish   WPSAKLLQAAAGASYRASLIGTQAVIPALLDQYNNRTQCAQRRTLLEVLQGFVQPTPLSR 420
Frog        WPSAKLLQAAAGGSSRACWKVTANVLPLLLEQYNQHAQSSHRRTILEMTLGFLKLQSRWL 420
                                      * * :**  :::.::***:**:   :::

Fugu        SS-EHGNLFLAFRPSLCSMVFSALVETNASLQITAASVLTLLAQQTGLLLDSDIELAVDH 479
Spotted     SP-ENGNLFLAFRPSLCSMAFSALVESNASLQITATSVLTLLARQTGLLLDSDVELAVDH 479
Medaka      STEDDDGALSAHRADLCGVAFSALSESDGSLQIAATALLASLAQQTGLLLESDVQLAADH 480
Stickleback S---EESVFSAFRPALCTVVFSALTETNSSLQITAASVLTSLARHSDLLLDSDIELAVDH 477
Smelt       SNMEDESVLSDFRASLCSVVFSALTESNAGLQVTATRVLTALAQHTGLLQQADVEQAVAH 480
Herring     PAEGEENVLLPFQSSLCGVVFSALSESGAGLQTTATRILTALGQQSGVLTPPDVDLAVDH 336
Zebrafish   PADGEESVLVAFQQSLCTVVFSALSETSAGLQVTATRVLTALSQQPGLLSQTDVENAVDH 480
Frog        DE-EDENGLGNLKESLCSMVFSAVTDSSTQLHVVALKILTVLAMQQGFMASSDIDLVVDH 479
                . . :   :  ** :.***: ::.  *: .*  :*: *. : ..:  .*:: .. *

Fugu        LTRLLLTEDDDRVSLAVVECVGALAELYPATFIAKLIPKLKTEIFSEVMDQDNNTVASEQ 539
Spotted     LTRLLLTEDDDRVSLAVVECVGALAELHPATFITKLLPRLKMEIFSEEMDQDNNTVVSEQ 539
Medaka      LTRLLLTEDDGRVSVAVVECAGVLAELHPAAFVTRLIPRLKEEMFS----------APQQ 530
Stickleback LTRLLLTEEDERVSLAVVECAGALAELRSAAFITNLIPRLKKELFSEPTDQKDNSEASEK 537
Smelt       LTRLVLGEADATVSVAVLDCAGSLAKLHPAAFITNMVPHLKDQIFTEPMALGDGSTLCQE 540
Herring     LTTLILQEDDPQVSGAVVEC???????????????????????????????????????? 356
Zebrafish   LTRLILEEEEAQVSLAVVECSGSLAHLHPHAFVSRMIPQLKEKILSDE------------ 528
Frog        LTRLILQKTDSESCMAAVEASGTLAKVHPSAFISRMLPQLCANLQTEPM--DINLNESGV 537
            ** *:* : :   . *.::.

Fugu        SSHHALRQRCMAALAMASTQPSVVLESTPVLLEVLSSAHTGSTRFSVAEVVLACHCLQKI 599
Spotted     SSHHALRRRCMSALAVSSTQASVVLESTPVLLEVLSSAHTGSARFSVDEVAMACRCLQRI 599
Medaka      HSHHALRRRCLSALAAVSTQPSVVQASTPVLLEVLKSALTGG--FSLEEAVLACRSLQRI 588
Stickleback LSHHAVRQRCLSALAAVSTQPGVVQESAPVLLEVLGSAHAGGVVFSVEEVVAACGSLRRI 597
Smelt       PSQHAVRQRCVSALAAVSSQPSVVQESTPVLLQVLSSAHTGSGCFSSEEVVSVCHSLQRI 600
Herring     ????????????ALARVACEPSLVQESTPVLLQVLTAAHTGGGAFSVDEVISVCHSLQRI 404
Zebrafish   -CSGAVRRRCVAALASVSSQPSVVQESSPVLLQVLTSAHTGCCGFSVEEVIAVCISLQRI 587
Frog        VPEHSIRQLCLEALAAVSTHQSILKETVPILLDYIRRAHNGEEETNVENVVSVCKSLHRV 597
                        ***  : . .::  : *:**: :  *  *    .  :.  .* .*:::
```

```
Fugu        AARA-QDTEETGRCFHDVIIPRLLCLALQAALRGEGPSDH-HSPLLEEAVLCAIVSVIST 657
Spotted     AEGA-QDTEETGRCFHDAVIPRLLCLALQAALRGEGASDP-HSPLLEEAVLCAVVSVIST 657
Medaka      AERV-QDTEEAGTCFHDVIIPRLLSLVLKAALQEEGSSGG---PLVQEDFLSAVVPVIST 644
Stickleback AERV-QDTEETGRCFHDVIIPRLLSLALQAALQGGGGSPGRRSPLLEEVVLAAMVPVIST 656
Smelt       AEQA-QDTEETSHIFHDIIIPRLLGLALQAALQGEGHAGP-RSPLVEESIISAMVPVIST 658
Herring     AEHAPHMEAEIGQFFHDVIIPRLLGLALQAAVQDEGPCG--RSPLVEEAVLSAVAPVISS 462
Zebrafish   AVHA-RDNEAIGQFFHDIIIPRLLGLTLQAALQSKDSGH--ISPLTDEAVLSAIVPVIST 644
Frog        AVQC-QLDSESLQFYHQTVLPCLLSLTVQAATQDSGTSS---HILLRDDILTAMVPVISA 653
            *    :         :*: ::* ** *.::** :  .        *  : .: *:..***:

Fugu        ACSRLQPSLAGQTASRAVSLFLDGDVSFLPDNSFPSHLQLLK---------PGDSWRQSQ 708
Spotted     ACSRLQPSLAGQTASRVVSLFLDGDVSFLPDNSFPSHLQLLKE-------ESGDSWRRSQ 710
Medaka      SCSRLQPTLAARTASRAISLFLDGDVSFLPENSFPSHLQLLQVEDPRLQKQPEVSWRRSQ 704
Stickleback SCSRLQPTLAGQTAARAVSLFLDGDVSFLPDNSFPSNLQLLKT-------QQGDSWSQSQ 709
Smelt       TCARLQPTLASQTASRAVSLFLDGDVSFLPDKAFPDHIQLLKK--------QSQYWSQSQ 710
Herring     CCSRLNADCAKLSASRAVSLFLNGDVAFLPNNNLPTNTQLLKT----------PSPQVSQ 512
Zebrafish   ACAALKPESASRMAAQAVSLFLDGDTSFLPENAFPSQIQPLQS--------QADSRGPSQ 696
Frog        ACTHLKPELASKSTSQIVSLFLDGDISLLSENNFSSKFQPFQV--------NGPTELQNR 705
             *: *:.  *   ::: :****:** ::*.:: :. : * ::                 .:

Fugu        LVCLLMACVCTLPRSVEVPQIDRLLSQLEEMSCTCSHQLSYSSAAKCFAGLVNKRPQGDS 768
Spotted     LVCLLMACVCALPRSVEVPQIERLLSQLEETSCTCSHQLSYTSAAKCFAGLVNKRPAGDS 770
Medaka      LVCLLMGCVCSLPRGVEVPRTEELLSRLEEMSCTCRHPPSYTAAAKCFAGLVNKSPEGDA 764
Stickleback MVCLLLGCVCSLPRTVEAPRLDELMTELEEMSCACSHPLSYTSAAKCFAGLANKRAAGES 769
Smelt       MVCLLMACVCSLPRSVEVPQMDRLLAELEELSCSNSHPLSYTSAAKCYAGLVNKAPPGDS 770
Herring     LVCLLTASVCSLPRSVEIPEIDRLLKDLEEVSCTHTHSFTHTFAAKCYAGLINKRPNGPA 572
Zebrafish   LVCLLMACVCSLPRSVEIPDMDRLLVQLEDLSCTSPHLFSYTFASKCIAGLVNKRPAGAA 756
Frog        LVSLLMAFICSLPRNVEIPHLRRLLQHLLSLSLSGCSLFAYSSASKCFAGLINKCPQGDL 765
            :*.** . :*:*** ** *   .*:  * . * :     ::: *:** *** ** . *

Fugu        LDRLIQGTMARVCSELDSTSSPLRSQAFTLMIWVAKALLLRYHPLITTLTDKLFSLLTDD 828
Spotted     LDGLIRGTMTRVCRELDSASSPLRTQAFTLIIWVAKALLVRYHPLSSTLTDKLFSLLNDD 830
Medaka      LDGLIQRTRRRICGELDS-SSPLRMQAFTLLVWVAKALLLRYHPLSAALTDQLFSLLDDA 823
Stickleback LDGLIQRTMKRVCSELDCPSSPARTQAFTLMIWVAKALLLRYHPLYATLTDKLFSLLDDA 829
Smelt       LESLIERTMKRVCSELDSPSSPQHTQALTLLMWVAKALLLRYHPLSTTLTDKLFSLLSDS 830
Herring     LDAVLERSMKRVSVELDNSASCLRTQALTLLLWVTKALLLRYHPLSTTLTDKLFSLLSDR 632
Zebrafish   LNAVLDRVLKRVSLELEETSSTHRTQAFTLLIWVAKALLLRYHPLSTALTDKLFSLLSDS 816
Frog        LDDILKVTAQRIDV--GLVEEPSRTQAITLLVWVTKALVLRYHPLSGQLTDKMIGLLSDQ 823
            *: ::     *:         .  : **:**::**:***::*****   ***:::.** *

Fugu        HLGLLAADGFSLLMTDSVDILNRSCHADVRIMYRQRFFSENSPKLVQGFNAARQEKKPNY 888
Spotted     QLGLLAADGLSLLMADSVDVLNRSCHADVRIMYRQRFFSENSAKLVQGFTEAPGEKKPNY 890
Medaka      DLGAPAADAFLLLMSDSPDVLNRGCHADVRLMYRQRFFSENSAKLVQGFNAAPQEKKPNY 883
Stickleback DLGPAAADAFLLLMSDTADILNRGCHADVRIMYRQRFFSENSAKLVQGFNAAPQEKKPNY 889
Smelt       ELGTSAADGFSLLMSDSVDVLNRGCHADVRIMYRQRFFTENSAKLVQGFNSAPPEKKSCY 890
Herring     ALGPLVADGFSLLMSDSSDVLNRSCHADVRIMYRQRFFSENSAKLVQGFNSAPPEEKAGY 692
Zebrafish   ALGSLVADGFCVLMNDSPDVLNRDCHADVRIMYRQRFFTENSSKLVQGFNSAEQAKKSCY 876
Frog        QLGPSVANMFSLLVSDSPDILNKACHADIRIMFRQRFFTENVPKLVQGFNSANGDDKPNY 883
             **  .*: : :*: *: *:**: ****:*:*:*****:** .******. *   .*. *

Fugu        LKALSNIVEKLPKQVQVTELPALLSLLLEALSCPDQGVQLSTLSCLQPVLVDPPPALIQQ 948
Spotted     LKALSNIVNKLPKQVQVTELPALLSLLLEALSCPDQGVQLSTLSCLQPVLVEPPPAVIQQ 950
Medaka      LKALSNIVNKLPKQVQVAELPALLSLLLEALTCPDQGVQLSTLSCLEPVIASPPPVLLQQ 943
Stickleback LKALSNIVNKLPKQVQLTELPALLSLLLEALLCPDHAVQLSTLSCLEPVLMEPPPALIQQ 949
Smelt       LKALSHIVNNLPRQVQLSELPALLPLLLEALSCPDQGVQLSTLSCLQPVLLDPPSALITQ 950
Herring     LKALSHIMNNLPREVQMTELPALLPLFLEALSCPDVGVQLSTLSCLQPVLLDPPSALTNQ 752
Zebrafish   LKALSHIVNNLPREVQLTELPALLPLLLEALSCVDQGVQLSTLSCLQPVLLESPAALNTQ 936
Frog        LKALSHVLNTLPKQVLMPELPSLLSLLLEALSCPDKVVQLSTLICLEPLLQEAPETLKVH 943
            *****::::.**::* :.***:**.*:**** * *  ****** **:*:: ..* .:  :
```

```
Fugu        LEALFSRLLALTSSPSMNMRIASLRCIKTISHFPVHEVLPFRARVLRALARPLDDRKRLV 1008
Spotted     LEALFSRLLTLTCSPSMNIRIASLRCIDAISHFPVHEVLPFRARVLRALAQPLDDRKRLV 1010
Medaka      LEAVVGRLLALVCSPAMEVRMSSLRCVRALSLLPTHQVLPFRARVLRALAPPLDDRKRLV 1003
Stickleback LEALVGRLLALTASAVMSVRIASLRCIHSISCFPAHEVLPFRARVLRALSRPLDDHKRLV 1009
Smelt       LEALVSRLLALTSSPAMKVRIASLRCVHAVSRFPEHEVMPFRTRVLRALAAPLGDNKRLV 1010
Herring     LESLIPRVLTLTTSRAMKVRITSLRCVHSLSRLPDHMLLPFRARVLRALAAPLDDKKRLV 812
Zebrafish   LEALFTRLLALTTSPAMKVRMASLRCVHALSRLPEHMVMPFRARVLKALAAPLDDKKRLV 996
Frog        IDGLISKLLGLSCSPAMAVRITALKCILALTKLPLHMLLPYKQQVIRALAKPLDDKKRLV 1003
            ::.:. ::* *  *  * :*:::*:*: ::: :* * ::*:: :*::**: **.*.****

Fugu        RQEAVQARAEWFLLGSPGGR 1028
Spotted     RREAVQARAEWFLLGSPGGR 1030
Medaka      RREAVQARGEWFLIGSPGGR 1023
Stickleback RREAVRARAEWFLLGSPGGS 1029
Smelt       RSEAVKARGEWFLLGSPGGR 1030
Herring     RKEAVLARGEWFLLGSPG-R 831
Zebrafish   RKEAVAARGEWFLLGSPGGR 1016
Frog        RKEAVETRCQWFLLGSPG-S 1022
            * *** :* :***:****
```

C

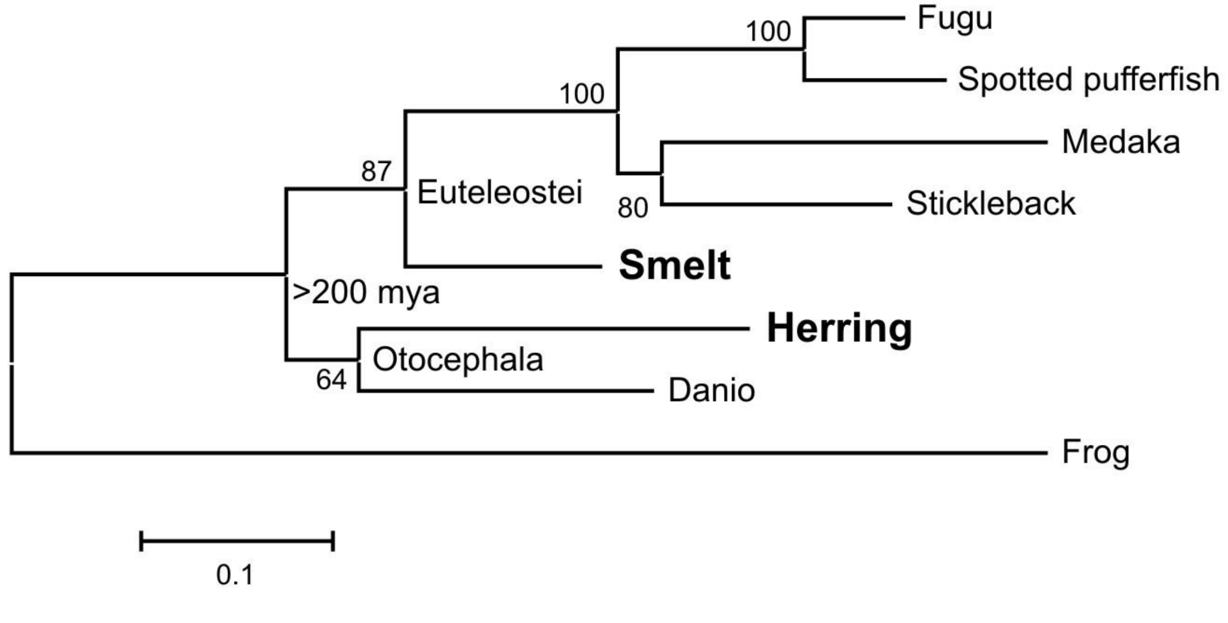

Supplement: Additional file 5 — Figure S2. Alignment of RBP3-2 protein sequences. [file 1471-2148-12-190-S5.pdf]
